# Supplementary material for: A model of conflict and leadership: Is there a hawkish drift in politics?
Source: PLoS One. 2022 Jan 14;17(1):e0261646. doi: 10.1371/journal.pone.0261646 (PMC8759682; doi:10.1371/journal.pone.0261646)
Supplement: S1 File — (PDF) [file pone.0261646.s001.pdf]

Supporting information

Appendix I Micro-Foundations and Theoretical Extensions

I.A We construct a simple model to provide micro-foundations for the expected cost of insurgency as a function of y and θ. We assume that the members of P have two choices - work, or join insurgency. The return to work are given by $\omega(y)$, which negatively depends on the land retained by C, i.e., $\omega(y) < 0$. Each P member also derives ideological benefits from joining the insurgency, which depends on his ”type” δ, denoted by $g(\delta)$ where $g(\delta) > 0$, where δ is distributed according the CDF $F()$.

A person of type δ will join insurgency if $g(\delta) \geq \omega(y)$ i.e. if

$$\delta \geq g^{-1}(\omega(y)).$$

Hence, the mass of P members joining insurgency is $1 - F(g^{-1}(\omega(y)))$. We assume that the potential cost of terrorist activity on C members is proportional to the size of the insurgent group.

We also assume that the politician in office is able to counter the insurgency and thereby defuse the cost (without any loss of generality, to 0) with probability $P(\theta)$ which is an increasing function of θ. Hence, the expected cost of insurgency is

$$[1 - P(\theta)] \cdot [1 - F(g^{-1}(\omega(y)))].$$

The specific example we used in our paper is $\omega(y) = 1 - \frac{y^2}{2}$, $F(\delta) \sim \mathcal{U}[0, 1]$ and $1 - P(\theta) = \frac{1}{1+\theta}$. Hence we have

$$C(y, \theta) = \frac{y^2}{2(1+\theta)}.$$

I.B In this part we check the robustness of our model to allowing for exogenous shocks to the cost of conflict to the society. A good shock means the expected cost of conflict is lower (either due to a lower probability of a conflict starting or a lower resultant damage from the conflict) while a bad shock means the expected cost is higher. Let $\lambda(\geq 0)$ denote the parameter which determines the level of the shock, whose

realized value is drawn from some probability distribution. Specifically, modify the voter's expected utility function in our baseline model by $U = u(y) - \frac{1}{\lambda} \cdot c(y, \theta))$ which, with our specific functional form used in the paper, is

$$y - \frac{1}{\lambda} \cdot \frac{y^2}{2(1+\theta)}.$$

There are three possibilities to consider:

1. The value of λ is unknown to the voter and the incumbent when choosing y. In this case the incumbent's ideal policy is $\arg\max y - E(\frac{1}{\lambda}) \cdot \frac{y^2}{2(1+\theta)}$, i.e.,

   $$y = \frac{1}{E(1/\lambda)}(1+\theta).$$

   As we can see, y is monotonically increasing in θ making it qualitatively similar to the baseline case, and therefore generates the same strategic considerations as studied paper since a higher y is a signal of higher incumbent ability.

2. Voter and incumbent know the value of λ before choosing y. In this case the incumbent's choice is contingent on the realized value of λ, and is $y = \lambda(1+\theta)$. Hence, a good (bad) shock makes the policies more hawkish (dovish). Also, knowing this, the voters will set a more hawkish re-election standard in good times and a lower one in bad times.

3. Incumbent knows the realized value of λ but the voter does not. In this case, as before, the incumbent's ideal policy is $y = \lambda(1+\theta)$. However, since voter can observe neither θ nor λ, screening is less precise. This is similar to the case of unknown incumbent ideology (α) that we examined in the paper. Here too voters will apply Bayes rule to infer θ from the observed y and based their re-election decision on this inference.

I.C In this part we check the robustness of our model to allowing the incumbent's ability to affect both the cost of conflict as well as management of the economy. To this end, we modify the voter's payoff function to be

$$U = \mu(\theta) \cdot [u(y) - \frac{c(y)}{\beta(\theta)}]$$

where $\beta(\theta)$ is the effectiveness of incumbent type θ in managing the conflict and $\mu(\theta)$ is the effectiveness of the leader in managing the economy (net of resources lost in conflict). Both $\mu(\cdot)$ and $\beta(\cdot)$ are strictly positive and weakly increasing in θ. Therefore, maximizing the U above is equivalent to maximizing the expression in the parenthesis as $\mu(\theta)$ acts as a shift parameter. In this case the results developed in the main section go through without any modification. This is because a higher ability leader has a greater net resource at his disposal, $u(y) - \frac{c(y)}{\beta(\theta)}$, and is able to generate a greater utility from better management of that resource.

An alternative formulation would be

$$U = \mu(\theta) \cdot y(\theta) - \frac{c(y)}{\beta(\theta)}.$$

In this case, the optimal policy choice is given by the condition

$$\frac{c'(y)}{u'(y)} = \mu(\theta) \cdot \beta(\theta).$$

Given the concavity of the $u(\cdot)$ function and the convexity of $c(\cdot)$, the LHS of the above equation is increasing in y while the RHS is increasing in θ, implying that a more able incumbent chooses a higher y. This leads to the same strategic considerations as studied in the baseline model since a higher y acts as a signal of higher incumbent ability.

Appendix II We provide here the values for the second best as well as the PBE re-election standard for various values of α and r. We also provide the corresponding values of θ_1 and θ_2 for the second best. These computations were performed using Mathematica as well as R. Code is available upon request.

a	r	W	$\bar{y}$	θ_1	θ_2	SBE=PBE?	$\hat{y}$
0.9	0	0.792	1.559	0.484	0.732	Yes	1.559
0.95	0	0.797	1.507	0.455	0.586	Yes	1.507
1	0	0.799	1.443	0.443	0.443	Yes	1.443
1.05	0	0.797	1.515	0.443	0.443	Yes	1.515
1.1	0	0.791	1.587	0.443	0.443	Yes	1.587
1.15	0	0.781	1.659	0.443	0.443	Yes	1.659
0.9	0.05	0.793	1.644	0.429	0.826	Yes	1.644
0.95	0.05	0.796	1.589	0.390	0.673	Yes	1.589
1	0.05	0.797	1.528	0.363	0.528	Yes	1.528
1.05	0.05	0.795	1.456	0.353	0.387	No	1.595
1.1	0.05	0.789	1.496	0.360	0.360	No	1.665
1.15	0.05	0.780	1.572	0.367	0.367	No	1.734
0.9	0.1	0.790	1.713	0.388	0.904	Yes	1.713
0.95	0.1	0.791	1.655	0.341	0.742	Yes	1.655
1	0.1	0.791	1.594	0.306	0.594	Yes	1.594
1.05	0.1	0.789	1.526	0.284	0.453	No	1.658
1.1	0.1	0.784	1.446	0.278	0.315	No	1.726
1.15	0.1	0.775	1.485	0.291	0.291	No	1.795
0.9	0.15	0.785	1.773	0.353	0.970	Yes	1.773
0.95	0.15	0.785	1.710	0.301	0.800	Yes	1.710
1	0.15	0.784	1.647	0.261	0.647	Yes	1.647
1.05	0.15	0.781	1.580	0.231	0.505	No	1.710
1.1	0.15	0.776	1.506	0.214	0.369	No	1.778
1.15	0.15	0.768	1.443	0.217	0.255	No	1.846
0.9	0.2	0.778	1.815	0.318	1.000	Yes	1.815
0.95	0.2	0.776	1.757	0.267	0.849	Yes	1.757
1	0.2	0.774	1.691	0.222	0.691	Yes	1.691
1.05	0.2	0.771	1.623	0.188	0.546	No	1.756
1.1	0.2	0.766	1.552	0.164	0.411	No	1.823
1.15	0.2	0.759	1.472	0.154	0.280	No	1.891
0.9	0.25	0.769	1.844	0.283	1.000	Yes	1.844
0.95	0.25	0.766	1.797	0.236	0.892	Yes	1.797
1	0.25	0.763	1.728	0.188	0.728	No	1.730
1.05	0.25	0.759	1.659	0.150	0.580	No	1.797
1.1	0.25	0.754	1.589	0.122	0.444	No	1.864
1.15	0.25	0.748	1.513	0.105	0.315	No	1.931
0.9	0.3	0.759	1.872	0.251	1.000	Yes	1.872
0.95	0.3	0.755	1.831	0.207	0.928	Yes	1.831
1	0.3	0.750	1.759	0.157	0.759	No	1.768
1.05	0.3	0.746	1.689	0.116	0.608	No	1.834
1.1	0.3	0.741	1.618	0.085	0.471	No	1.901
1.15	0.3	0.735	1.545	0.063	0.343	No	1.968
0.9	0.35	0.748	1.898	0.222	1.000	Yes	1.898
0.95	0.35	0.742	1.861	0.180	0.959	Yes	1.861
1	0.35	0.737	1.785	0.127	0.785	No	1.802
1.05	0.35	0.732	1.713	0.084	0.632	No	1.869
1.1	0.35	0.727	1.642	0.051	0.493	No	1.936
1.15	0.35	0.721	1.570	0.026	0.365	No	2.003
0.9	0.35	0.748	1.898	0.222	1.000	Yes	1.898
0.95	0.4	0.728	1.886	0.154	0.985	Yes	1.886
1	0.4	0.723	1.807	0.099	0.807	No	1.834
1.05	0.4	0.717	1.000	0.000	0.000	No	1.901
1.1	0.4	0.712	1.000	0.000	0.000	No	1.968
1.15	0.4	0.706	1.000	0.000	0.000	No	2.035
